# Molecular Insight into the Effect of Polymer Topology on Wettability of Block Copolymers: The Case of Amphiphilic Polyurethanes

**DOI:** 10.1021/acs.langmuir.3c01646

**Published:** 2023-12-15

**Authors:** Alireza Mirzaalipour, Elnaz Aghamohammadi, Helma Vakili, Mohammadreza Khodabakhsh, Ugur Unal, Hesam Makki

**Affiliations:** † Department of Polymer and Color Engineering, Amirkabir University of Technology, 424 Hafez Ave., 159163-4311 Tehran, Iran; ‡ Polymer Engineering group, School of Chemical Engineering, College of Engineering, University of Tehran, 1417935840 Tehran, Iran; § Chemistry Department, Koc University, Rumelifeneri Yolu, Sariyer 34450 Istanbul, Turkey; ∥ Koc University Surface Science and Technology Center (KUYTAM), Koc University, Rumelifeneri Yolu, Sariyer 34450 Istanbul, Turkey; ⊥ Department of Chemistry and Materials Innovation Factory, University of Liverpool, Liverpool L69 7ZD, U.K.

## Abstract

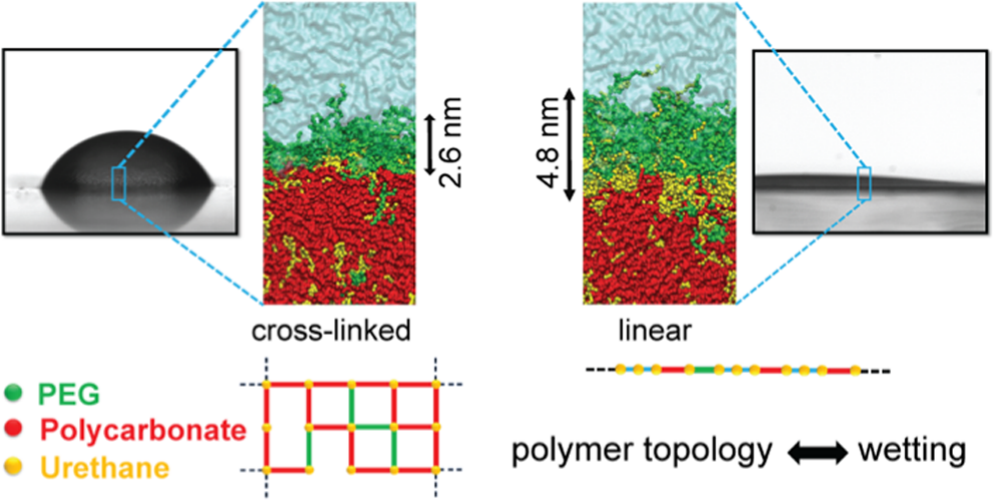

The microstructure design of multiblock copolymers is essential for achieving desired interfacial properties in submerged applications. Two major design factors are the chemical composition and polymer topology. Despite a clear relationship between chemical composition and wetting, the effect of polymer topology (i.e., linear vs cross-linked polymers) is not very clear. Thus, in this study, we shed light on the molecular origins of polymer topology on the wetting behavior. To this end, we synthesized linear and three-dimensional (3D) cross-linked network topologies of poly(ethylene glycol) (PEG)-modified polycarbonate polyurethanes with the same amount of hydrophilic PEG groups on the surface (confirmed by X-ray photoelectron spectroscopy (XPS)) and studied the wetting mechanisms through water contact angle (WCA), atomic force microscopy (AFM), and molecular dynamics (MD) simulations. The linear topology exhibited superhydrophilic behavior, while the WCA of the cross-linked polymer was around 50°. AFM analysis (performed on dry and wet samples) suggests that PEG migration toward the interface is the dominant factor. MD simulations confirm the AFM results and unravel the mechanisms: the higher flexibility of PEG in linear topology results in a greater PEG migration to the interface and formation of a thicker interfacial layer (i.e., twice as thick as the cross-linked polymers). Accordingly, water diffusion into the interfacial layer was greater in the case of the linear polymer, leading to better screening of the underneath hydrophobic (polycarbonate) segments.

## Introduction

Chemical composition and surface morphology are two important factors determining the surface properties of polymer coatings such as wettability, a critical surface property for a wide range of applications, including anti-icing,^1,2^ anticorrosion,^2,3^ antisoiling,^4,5^ antibacterial,^6,7^ and antibiofouling coatings.^8−11^ Surface morphology and composition might differ from the bulk due to the surface rearrangements of polymer chains.^12−15^ This is an important characteristic of soft and multiblock polymer coatings, which provides a spontaneous response to the interacting environment to minimize the interfacial free energy.^16^ The wettability of a polymer surface can be generally characterized by water contact angle (WCA) measurement, which is a highly informative and easy-to-measure surface analysis technique.^17,18^ However, due to the rearrangement of the surface of soft multiblock polymers in contact with water and water diffusion into the polymer film, particularly in the case of polymers containing hydrophilic blocks,^19^ the WCA may vary over time, thus, it is often infeasible to describe wettability for polymer surfaces with a single static WCA value.^20^

Time-dependent WCA has been widely used to characterize polymer surface rearrangements,^12,21−24^ however, the exact underlying mechanisms of time-dependent WCA are still unclear for many cases.^25,26^ There are a number of studies that correlated the time-dependent WCA with morphological changes in polymer surfaces. For instance, changes in the WCA of plasma-treated nylon were explained by the rearrangement of the surface structure due to the strong interaction between water and nylon, which was concluded to be due to the surface reconstruction of the treated polymer.^20^ The origin of this phenomenon was described as the local deformation of the soft solid surface and the related viscoelasticity dissipation by Shanahan and Carre.^21^ In another study, Li et al. rationalized the time-dependent decrease in the WCA of poly(*N*-isopropylacrylamide)-grafted polypropylene films by grafting layer wetting. They discovered that, below a specified critical temperature, the interaction between the probing water and the film surfaces resulted in a decrease in the WCA during the measurement.^22^ In this line, Inutsuka et al. performed time-dependent WCA on amphiphilic diblock copolymers containing poly(dimethylsiloxane) (PDMS) and poly(ethylene glycol) (PEG) and they attributed the temporal reduction of WCA to the dynamic polymer brush formation through the segregation of amphiphilic copolymer toward the polymer–water interface.^23^ Also, in similar studies performed by Pike et al.^24^ the surface rearrangements of linear poly(dimethylsiloxane-urea-urethane) copolymers were monitored by WCA. They justified the time-dependent changes in WCA by the migration of polar hard blocks to the interface due to their affinity to water. Recently, Vakili et al. synthesized smart polyurethanes (PUs) based on (hydrophobic) polycarbonate (PC) and (hydrophilic) PEG soft segments, and their antibiofouling performance was demonstrated after a thorough investigation of the surface behavior.^27^ Time-dependent changes in WCA were shown to be caused by reversible phase arrangements of the polymer through experimental measurements and molecular dynamics (MD) simulations.^12^

Another key parameter has been recently found to have a significant impact on time-dependent wettability and interfacial properties: the polymer topology.^28−30^ For instance, Divandari et al. introduced the effect of polymer topology on the interfacial properties of poly(2-ethyl-2-oxazoline) (PEOXA) brushes on TiO_2_ substrates, i.e., protein adsorption. They demonstrated how switching the grafted chains’ topology from linear to cyclic allowed precise control of the interfacial and physicochemical characteristics of polymer surfaces without the need for intolerable fabrications or complicated chemistries.^28^ In another work, Morgese et al. showed how changing the topology of polymer brushes (with similar chemical compositions) from linear to cyclic, considerably enhanced the lubricity and steric interactions between polymer-grafted inorganic nanoparticles, without employing tedious fabrications and involving complex chemistry.^29^ Later, Albers et al. synthesized hydrophobic poly(propylene glycol) networks modified with hydrophilic elastically active network chains (PEG) (which were connected to the network from both sides) and monomethyl ether (mPEG) as hydrophilic dangling chains. They showed that mPEG-modified networks demonstrate better wet lubricity and correlated this to the formation of a strong hydration layer for these coatings.^31^ In a more comprehensive work, Butt et al. modeled the adoptive wetting behavior (i.e., reversible reconstruction of polymer surfaces at the interface with liquids) of a great variety of polymer surfaces.^32^ They recognized such behavior with the dynamic contact angle phenomena and contact angle hysteresis of those materials. Also, a similar interfacial behavior has been studied by Saito et al.^33^ They showed that imposing confinement on elastomeric polymer brushes (e.g., by fixing them on a substrate) can decrease the level of response of polymers to the contacting liquid. Nevertheless, despite the proven significant impact of polymer topology/constraint on interfacial properties at polymer–liquid interfaces, the mechanisms of its effect on WCA and its time-dependent changes have not been provided clearly thus far. Therefore, in this study, we try to shed light on this phenomenon and elucidate its underlying mechanisms on a molecular scale.

In previous research,^34,35^ linear multiblock PU coatings based on (hydrophobic) PC and (hydrophilic) PEG were synthesized, which showed superhydrophilic behavior by incorporating only a small amount of PEG (≥20 wt % PEG/PC) while the water uptake of the coatings was maintained below 12 wt %. The term superhydrophilic refers to surfaces on which water is completely spread and has a contact angle of less than 5°.^34,35^ It was found that the superhydrophilic behavior is due to the migration of PEG blocks toward the interface and covering the polymer–water interface, and the surprisingly low water uptake was due to the PC segments that blocked water from further penetrating to the bulk of the coatings. In this work, both linear and cross-linked three-dimensional (3D) network analogous coatings are studied so that we can distinguish the contribution of polymer topology (i.e., linear vs cross-linked) on the wetting process (characterized by WCA). Thus, in this paper, we show that coatings with similar surface chemistry (proved by X-ray photoelectron spectroscopy (XPS)) but with different chain confinements (i.e., free linear chains from both ends vs chains confined in a 3D cross-linked network) have significantly different WCAs. This significant difference in wetting has molecular origins at the interface layer; therefore, we employ coarse-grained (CG) MD simulations to elucidate the surface rearrangements of polymers on the molecular scales as they are exposed to a water layer. By combining experimental and simulation approaches, we can demonstrate how coatings with similar chemical compositions can exhibit distinct wet morphologies and accordingly markedly different wetting behaviors. Therefore, the underlying mechanisms are explored, and based on that, possible superhydrophilic coating design strategies are discussed.

## Experimental and Simulation Methods

### Materials

Polyhexamethylene carbonate diol (PC) (*M*_w_ = 2000 g/mol, UBE corporation, Japan) and poly(ethylene glycol) (PEG) (*M*_w_ = 2000 g/mol, Merck) were dried under vacuum for 24 h at 70 °C. Desmodur N-75 (aliphatic polyisocyanate based on hexamethylene diisocyanate (HDI), Perstorp, Sweden) was used as the cross-linking agent. 1,4-Butanediol (BD) was obtained from Sigma-Aldrich, distilled, and dried over 4 Å molecular sieves. Methyl ethyl ketone (MEK, Merch) as the solvent and dibutyltin dilaurate (DBTDL) (MEK, Merch) as the catalyst were used as received.

### Synthesis

The NCO/OH ratio and the final solid content of all formulations were kept at 1.05 and 30%, respectively. First, the calculated amounts of polyols (PC and PEG) and the required amount of solvent (MEK) were poured into a vial, and then the mixture was heated in a silicone oil bath until it was completely homogeneous and clear. The oil bath temperature was preset to 70 °C. Then, 5 wt % solution of DBTDL in MEK (0.02 wt % of the total formulation) was injected into the mixture. In the next step, the calculated amount of the cross-linker was added to the polyol solution, and the reaction was allowed to continue at 70 °C. As the viscosity of the solution slightly increased, the synthesis vial was removed from the bath, and the final solution was spin-coated (at 1000 rpm for 30 s) onto glass slides. An hour after application, the slides were placed in a convection oven at 70 °C for 24 h. The details of the synthesis of the linear polymer are covered in the preceding paper.^12−15^ However, for this study, the linear polymer (after synthesis) was dissolved in MEK (30% solid content) and spin-coated (at 1000 rpm for 30 s) onto glass slides, similar to the cross-linked samples. All sample formulations are shown in Table 1 (cross-linked) and Table 2 (linear).

**Table 1:** Formulation of Cross-Linked Samples

sample	PEG (g)	PC (g)	Desmodur N-75 (g)	5 wt % solution of DBTDL (g)
PU–PEG0	0	2	0.534	0.012
PU–PEG10	0.2	1.8	0.534	0.012
PU–PEG20	0.4	1.6	0.534	0.012
PU–PEG30	0.6	1.4	0.534	0.012

**Table 2:** Formulation of Linear Samples

sample	PEG (g)	PC (g)	HDI (g)	BD (g)	1 wt % solution of DBTDL (g)
PU–PEG 0	0	2	0.504	0.18	0.266
PU–PEG10	0.2	1.8	0.504	0.18	0.266
PU–PEG20	0.4	1.6	0.504	0.18	0.266
PU–PEG30	0.6	1.4	0.504	0.18	0.266

### Characterization

For linear samples, the number-average molar mass (*M_n_*), weight-average molar mass (*M*_w_), and dispersity index (DI) for linear samples were determined using gel permeation chromatography (GPC) performed on a PL-GPC 50 (Agilent Technologies). Polystyrene standards were used to calibrate the equipment, and DMF (1 mL/min) was used as the solvent in GPC. GPC results are provided in the SI (Table S1).

Fourier transform infrared (FTIR) spectra of the coatings were recorded in attenuated total reflection (ATR) mode using a Nicolet iS10 spectrometer (Thermo Fisher Scientific, Waltham, MA) with 16 scans at a resolution of 4 cm^–1^ over a range from 400 to 4000 cm^–1^.

X-ray photoelectron spectroscopy (XPS) measurements were performed using a Thermo Scientific K-α spectrometer (Thermo Fisher Scientific, Waltham, MA) with a monochromatized Al Kα X-ray source (spot size ∼400 μm) at a takeoff angle of 90° to determine the elemental composition at the surface of the coatings. Binding energies for the XPS spectra were calibrated by setting C 1s binding energy to 284.5 eV. For fitting the deconvoluted peaks, “Thermo Avantage” software (Version 5.97) was used, and the peaks were fitted by a Gauss–Lorentz product function and a “Simplex” fitting algorithm.

Surface topography and phase images of the specimens were examined by atomic force microscopy (AFM) (Bruker, Dimension Icon) in the tapping mode, and an antimony-doped silicon microcantilever probe with a force constant of 40 N/m (Bruker) and a resonance frequency of about 300 kHz was used.

Static water contact angle (SCA) measurements were performed by the sessile drop method with a Jikan CAG-20 instrument (Iran) using deionized water as a probe liquid. The contact angle of the coatings was determined using JIKAN assistance software and the Low Bond Axisymmetric Drop Shape Analysis (LB-ADSA) plugin of ImageJ software. For measuring the static contact angle, a 5 μL water droplet was placed on the sample. An average contact angle was calculated from at least three droplets, and the standard error was reported. It should be noted that throughout the experiment, the temperature and humidity remained constant at 25 °C and 36%, respectively.

Differential scanning calorimetry (DSC) experiments were performed using a DSC 214 Polyma (NETZSCH, Germany), from −100 to 150 °C at a constant heating rate of 10 °C min^–1^ under a nitrogen flow.

### Simulations

**Figure 1:**
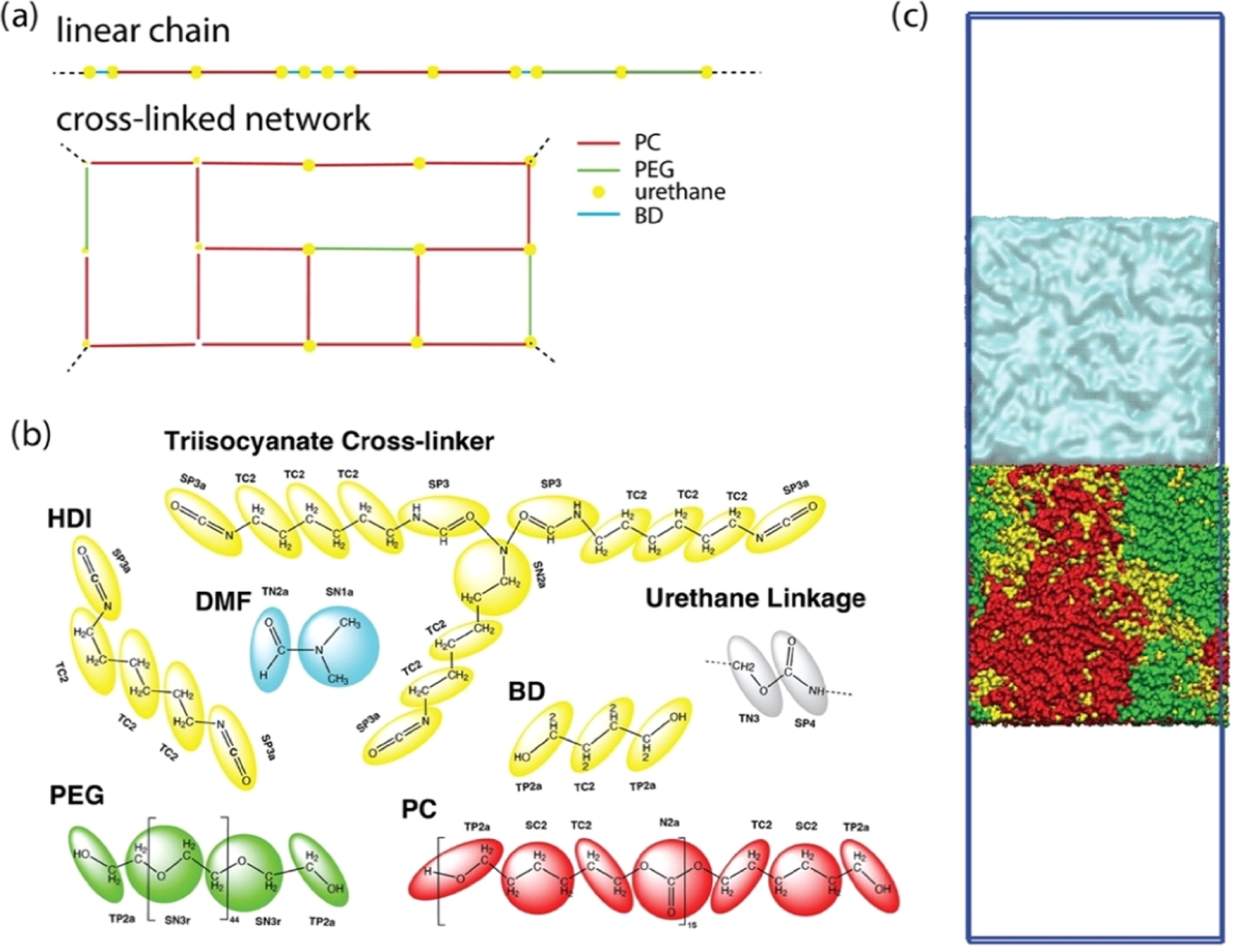
(a) Schematic presentation of linear and cross-linked topology, (b) bead typing based on Martini 3 standard beads, and (c) initial configuration of the polymer/water interface model (the box is chosen large enough to ensure that the polymer layer contacts water only on one surface).

MD simulations were performed using the GROMACS package.^36,37^ Using our recently developed MD-based polymerization code (PolySMart),^38^ based on Martini 3 coarse-grained representation^39^ we constructed linear multiblock and 3D cross-linked network PUs (see Figure 1a). Note that atomistic simulations were employed to determine the CG bonded parameters using the OPLS-AA force field,^40^ see the Supporting Information (SI), Section S2, and the nonbonded interaction parameters (σ and ε) for each pair of beads (group of atoms) were based on standard Martini 3 bead typing (see Figure 1b). A robust verification scheme has been performed for each material so that the density, radius of gyration, and end-to-end distance were compared with experimental and atomistic simulation data to validate the CG models; see the SI (Table S4).

Linear multiblock polymers and cross-linked networks were generated under the same conditions and sequences as those used in the experiment through our polymerization simulation scheme. The difference between the two topologies is presented in Figure 1a. For linear PUs, according to our previous study,^27^ diol and HDI were mixed and reacted in the presence of a model solvent (50 wt % solid content). In the next step, BD was added to the simulation box, and the reaction continued until the conversion reached above 98%. For cross-linked PUs, diols and crosslinker were mixed at a 1.05:1 ratio of NCO:OH, then reacted in the presence of 50 wt % of a model solvent. this was followed by energy minimization by a steep integrator and equilibration under *NPT* conditions at 300 K and 1 bar with a 10 fs time step for 5 ns. A Parrinello–Rahman barostat^41^ and the V-rescale thermostat^42^ were used for *NPT* simulations. Then, the reaction between hydroxyl and isocyanate beads was switched on. This step was repeated until more than 99% of the OH beads reacted.

*M_n_*, *M*_w_, and DI of linear samples were calculated for simulated polymer chains by a method developed in our previous work,^43^ see Table S5. Accordingly, molecular weights between cross-links (*M*_c_) and cross-link density for cross-linked samples were calculated and are summarized in SI Table S6.

After reaching the desired reaction conversions, we further equilibrated the polymers through an *NPT* simulation with a time step of 20 fs for 200 ns at 300 K and 1 bar (a thermostat and a barostat similar to those above) to equilibrate the PU models. To analyze the water–polymer interface, an equilibrated Martini 3 water layer with a thickness of 10 nm was placed in contact with the PU models. Note that simulation boxes for the polymer/water interface have been large enough to ensure that the water layer has no effect on the polymer layer through the periodic boundary; see Figure 1c. Then an *NVT* equilibrium was performed for 200 ns employing the V-rescale thermostat^42^ at 300 K with a time step of 20 fs. The density profile was employed to study the segregation dynamics of the polymer and the partial diffusion of water to the interface. We also investigate the hydrophilic segment migration to the water Interface using the numerical integral of the density profile. Water and polymer bulk boundaries were determined by fitting a sigmoid function to the density profile of water. As a result, the water–polymer interface thickness and hydration degree of the interface were calculated.

## Results and Discussion

### Synthesis Verification

ATR-FTIR was performed to verify the synthesis and characterize the chemical structures of PU samples. According to the complete band assignment^44^ and spectra of all samples (given in the SI, Figure S1), the representing peak of NCO functional groups at 2270 cm^–1^ disappeared, indicating that all isocyanate groups have undergone a chemical reaction. Also, the stretching vibration of NH at 3320 cm^–1^, C=O at 1680 cm^–1^, and bending vibration of NH at 1540 cm^–1^ proved the formation of urethane. The appearance of the C–O–C ether vibration at 1110 cm^–1^ for all PEG-containing samples is a clear indication of having PEG segments in the polymer structure. Furthermore, the intensity of peaks at 1740 cm^–1^ related to C=O confirms the presence of PC in all samples.^44^

### Wetting Behavior

**Figure 2:**
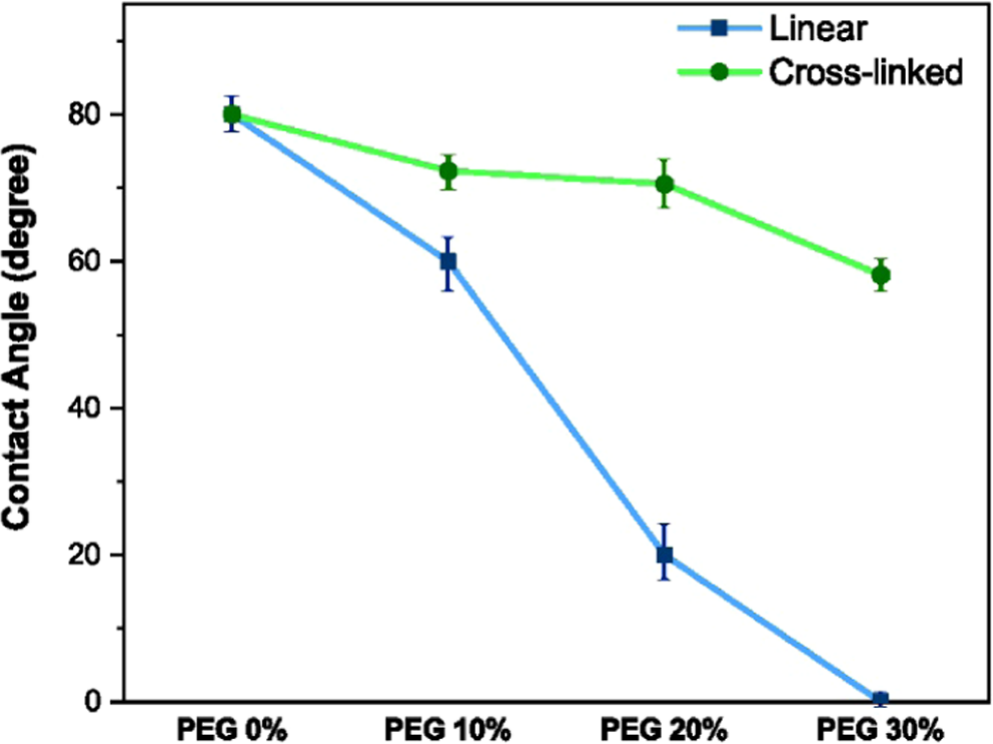
Static water contact angle (WCA) for different PEG contents for cross-linked and linear topology PUs.

The static WCA values are depicted in Figure 2 for cross-linked and linear PUs. As shown, for both polymer topologies, the WCA of the PEG-free samples (PEG 0%) is about 80° and as the PEG content increases, the WCA decreases. Also, the slope of the change in the WCA of linear and cross-linked samples markedly differ so that despite the similar PEG 0% WCA, the difference between WCA of cross-linked PEG 30% and linear PEG 30% polymers is about 50°. In other words, PEG 0% and PEG 30% (linear and cross-linked) samples show the lowest and highest levels of difference in wettability, respectively. Thus, we selected these formulations (i.e., PEG 0 and 30%) for both linear and cross-linked topologies for further analysis of the effect of polymer topology on the wetting mechanisms.

### Surface Chemistry of Dry Films

**Figure 3:**
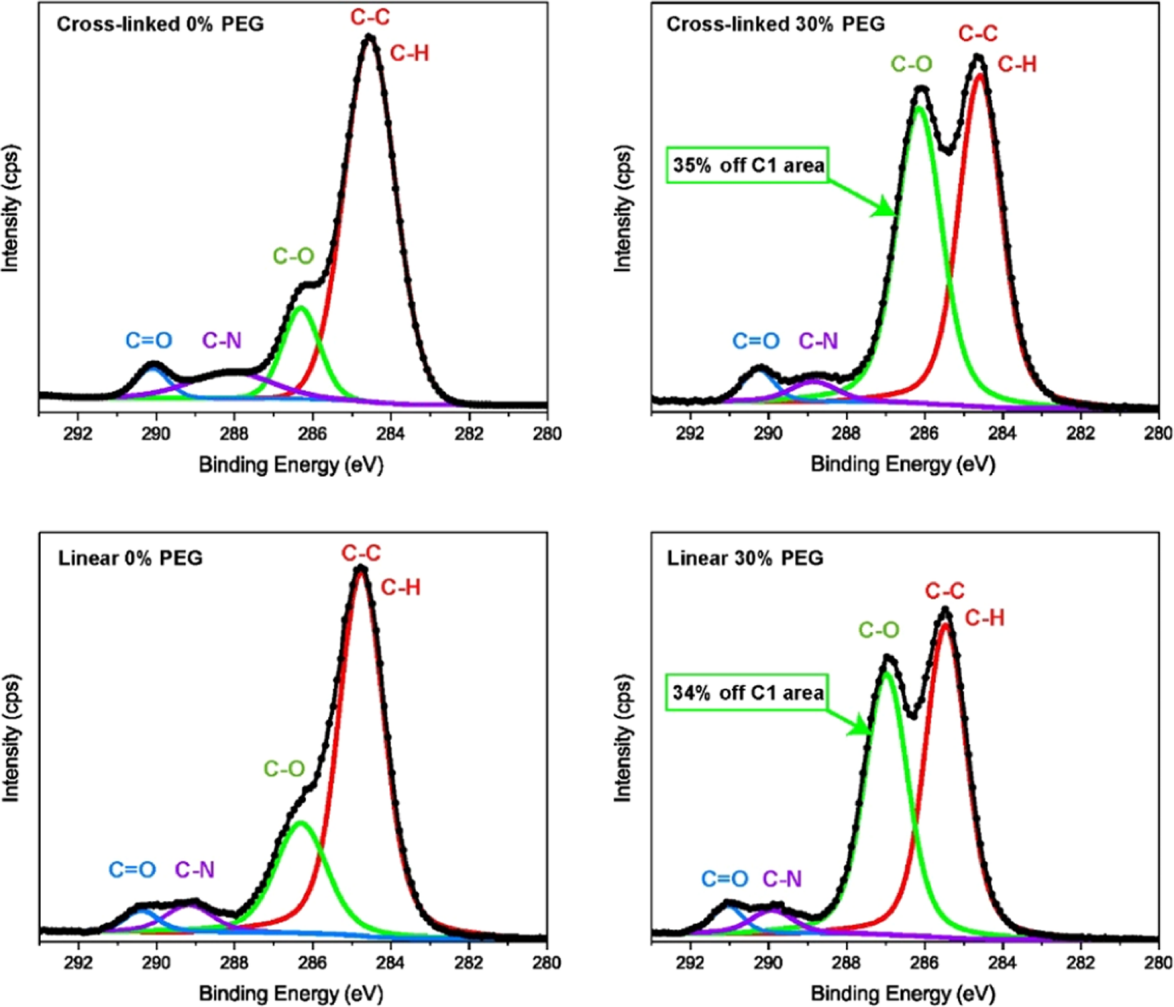
Deconvolution of XPS high-resolution C 1s spectra. The percentage of ether group (C–O) is shown for PEG-containing samples. The deconvoluted peaks were fitted by a Gauss–Lorentz product function using a Simplex fitting algorithm.

First, we need to confirm the similarity of surface chemistry of the linear and cross-linked (PEG 0 and 30%) samples in the dry state. Therefore, we used XPS to obtain quantitative information about the surface elements and functional groups of the two topologies. Note that XPS survey scans are provided in the SI (Figure S3) and the atomic percentages of the surface elements (calculated from survey scans) are presented in Table 3. The data shown in Table 3 clearly suggest that the number of different elements present on the dry surface of the samples is similar despite the significant difference in the topology of polymers. There is a slight difference in the N 1s peak of the linear and cross-linked samples, which is due to the greater number of urethane groups due to different structures and functionality of isocyanates as shown in Figure 1. As illustrated in Figure 3, the deconvoluted high-resolution C 1s spectra of the samples verify the quantitative similarity of functional groups at the surfaces. In Figure 3, the presence of C–C (∼285.5 eV), C–O (∼287 eV), C–N (∼289 eV), and C=O (∼290 eV)^45,46^ bonds are clear. A comparison between the atomic % for the deconvoluted carbon peak for C–O (35% for cross-linked and 34% for linear sample, as shown in Figure 3) confirms that the amounts of PEG segments at the surface of both polymer topologies are very similar in the dry state. Thus, one can ensure that the significant differences in wettability (as observed by WAC measurements) are most likely due to different interfacial rearrangements of polymers at the polymer/water interface during the wetting process.

**Table 3:** XPS Atomic Percentage Results Obtained from the Survey Scans Are Shown in Figure S1 in the SI

	atomic %		
sample	C 1s	N 1s	O 1s
cross-linked 0% PEG	73.81	1.84	24.35
linear 0% PEG	70.94	4.91	24.15
cross-linked 30% PEG	67.46	2.24	30.3
linear 30% PEG	68.09	2.82	29.09

We also performed DSC measurements to evaluate the bulk phase organization of linear and cross-linked samples. For the linear polymers, a clear phase separation between PEG and PC was recognized by observing two distinct melting peaks in the DSC thermograms as illustrated in our previous work.^27^ The thermogram of the linear PEG 30% samples shows two endothermic melting (*T*_m_) peaks at about 27 and 44 °C, close to the melting points of PEG and PC compounds.^27^ The appearance of two endothermic melting peaks indicates the presence of segregated crystalline domains of PEG and PC. For cross-linked polymers, no melting peaks have been observed. Nevertheless, two glass transition temperatures (*T*_g_) at 50.6 °C and −47 °C were found for the PEG 30% sample, which are (most likely) associated with hard and soft segments *T*_g_s. Additionally, the *T*_g_ of 30% PEG is 25 °C lower than that of 0% PEG, which results in higher molecular mobility. The thermograms of the 0 and 30% PEG samples and the calculated melting points and *T*_g_s for both topologies are given in the SI (Figures S4 and S5 and Table S7).

### Surface Morphology Changes upon Wetting

The change in the surface topography of linear and cross-linked samples, as a result of exposure to water, has been evaluated by AFM. Table 4 shows the *R*_q_ and *R*_a_ values for the wet and dry samples. Note that the wet sample measurements were performed by immersing samples in water for 24 h, quickly wiping their surface with a lint-free cloth, and a mild flow of nitrogen gas, prior to the measurement. The AFM images (topography and phase) for dry and wet cross-linked and linear samples are provided in Figures S6 and S7, respectively, in the SI. It should be noted that AFM phase images have been widely used to elucidate the change in the hard and soft segment organization for PU coatings^47−50^ as it has been shown that mechanical properties of different segments are the primary cause of the phase angle shift.^47^ However, we are interested in the change in PEG and PC surface concentrations (which both form the soft segment of the coatings and have similar mechanical properties) during wetting, and one cannot expect that the AFM phase image analysis will unravel such a surface morphological change. Therefore, our major focus is on the change in the surface roughness, as obtained from AFM topographic images. We observe that after immersing the samples in water, the surface roughness of cross-linked and linear PEG 0% samples remains almost unchanged (see Table 4). In the case of PEG 30% samples, the roughness increases for both topographies similarly (about 50% increase for both cases) as a result of exposure to water. The increase in surface roughness in the case of PEG-containing samples is probably due to the migration of PEG segments toward the surface and/or diffusion of water into the very surface layers of polymers, which are enriched with PEG due to the presence of polar water molecules at the interface.

**Table 4:** Roughness Values of PUs before and after Water Immersion

	PEG 0%		PEG 30%	
cross-linked	*R*_q_ (nm)	*R*_a_ (nm)	*R*_q_ (nm)	***R***_a_ (nm)
before water immersion	19.5	17.0	33.2	26.1
after water immersion	19.1	15.8	49.9	39.5
linear	*R*_q_ (nm)	*R*_a_ (nm)	*R*_q_ (nm)	***R***_a_ (nm)
before water immersion	16.8	13.7	24.5	18.9
after water immersion	17.3	14.4	37.1	29.6

So far, the experimental data suggested that PEG migration toward the surface as a result of wetting is a likely scenario for both polymer topologies. The amount of this migration can possibly be different due to the limitation in the mobility of polymer segments under different topological constraints (i.e., PEG segments in linear topology have higher mobility). However, PEG migration toward the surface cannot be the only explanation for the wetting behavior that we observed. One should note that the WCA of the PEG monolayer is around 36 degrees,^51^ not zero. Thus, even a 100% enrichment of the surface with PEG cannot result in a superhydrophilic surface, as observed in the case of linear polymer containing 30 wt % PEG. An additional likely mechanism can be water penetration through the very top layers of polymers. The thickness of layers enriched with water can be different for different polymer topologies (due to different amounts of accessible hydrophilic segments at the interface for linear and cross-linked systems), resulting in considerably different WCAs, see Figure 2.

### MD Simulations of the Wetting Process

For further justifications for the (unexpectedly) zero WCA of the linear sample, containing 30% PEG, as well as the mechanisms behind the significantly different wetting behavior of cross-linked and linear polymers, we performed MD simulations of the wetting process. Through MD simulations, we can directly see how water interacts with the polymer surface and how the phase organization of polymer surfaces changes immediately after exposure to water on a molecular scale. In this way, we can justify the likely scenarios suggested by the experimental measurements.

We created 10 nm-size polyurethane boxes with linear and cross-linked topologies using polySMart^38^ under exactly similar conditions to those in experimental synthesis. Note that we only simulated the PEG 30% samples as we are interested in PEG migration toward the surface and water penetration into the polymers. By inserting a water layer with a thickness of 10 nm onto the polymer surfaces and running MD equilibration, the nanophase organization of polymers was depicted and quantified.

**Figure 4:**
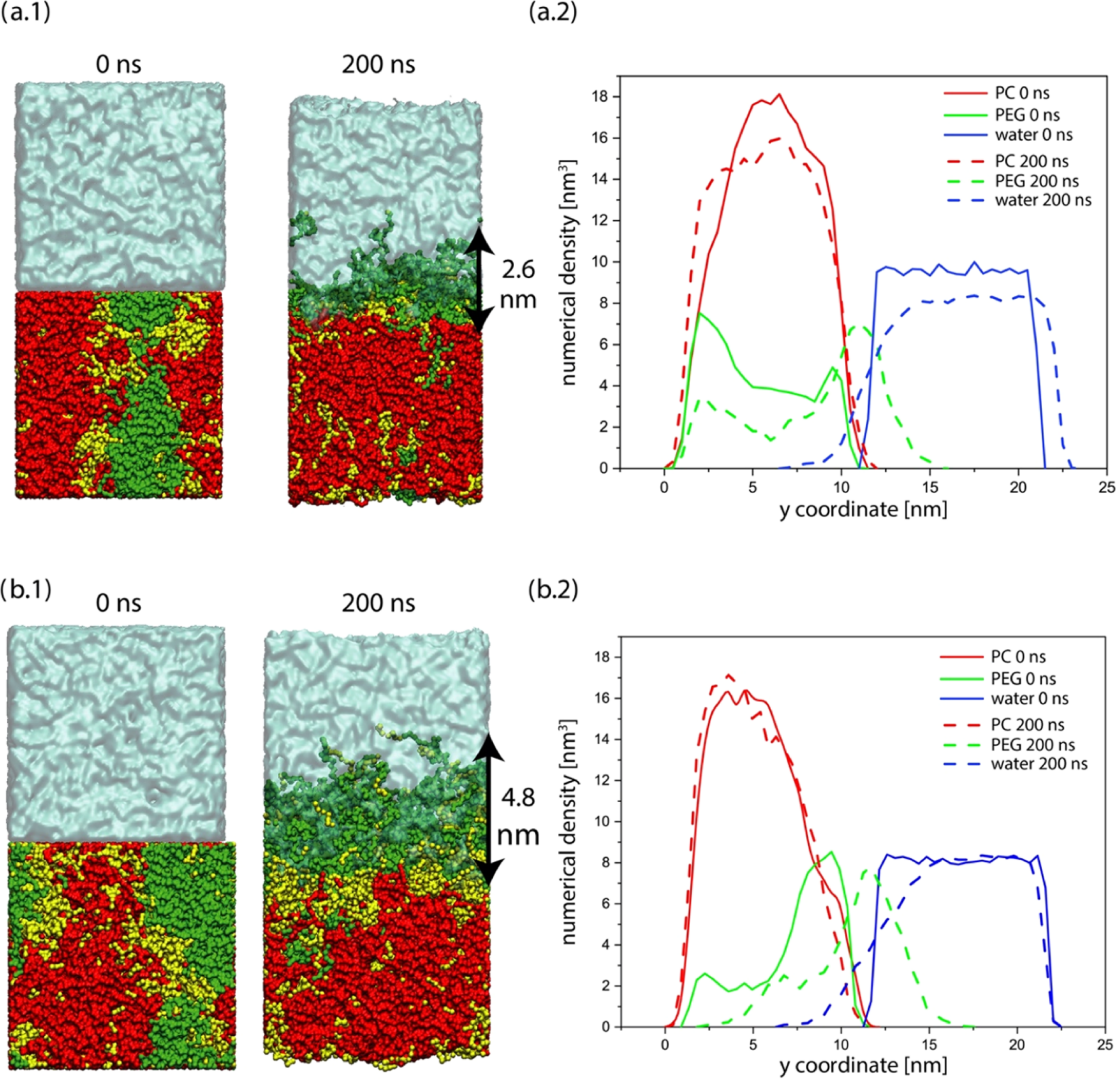
Side view of cross-linked (a.1) and linear (b.1) PU boxes before and after exposure to water. PC, PEG, and urethane segments are shown in red, green, and yellow, respectively. The distinct phase separation between PEG and PC phases and the formation of a large number of urethane groups at the interface between PEG and PC phases are discussed in (27) detail. Numerical density profiles of cross-linked (a.2) and linear (b.2) PU boxes.

The side views of the simulation boxes at the beginning (0 ns) and the end (200 ns) of polymer/water equilibration simulations are shown in Figure 4. The numerical density profiles of PEG segments, PC segments, and water for the dry (beginning of simulations) and wet (end of the simulations) samples are shown in Figure 4a.2, and b.2. As anticipated, a considerable portion of total PEG segments (72% for linear and 35% for cross-linked cases) in the simulation box migrates to the interface when the polymer surface is exposed to water. The lower amount of PEG migration for cross-linked polymers supports the idea of restricted mobility of hydrophilic segments in the cross-linked network. Note that these values were calculated by integrating the PEG density profiles for simulation snapshots taken at 0 (dry state) and 200 ns (wet state). The simulation result also shows that besides PEG segments, urethane groups tend to move toward the interface probably due to their higher polarity as compared to PC segments. Numerical density profiles of urethane groups are given in the SI (Figure S8) for dry and wet state. Again, urethane group migration toward the interface is more pronounced in the case of linear samples, indicating the restricted mobility of chains in the cross-linked topology. Furthermore, the density profiles quantitatively show that the surface has considerably rearranged to become more hydrophilic, which is an expected behavior to reduce the interfacial tension. Another interesting observation is that water molecules only partially reach the polymer surface layers and do not penetrate the bulk of the polymers. This finding is in line with the low water swelling seen in the earlier study for the linear samples,^12^ which is due to the hydrophobic nature of PC segments underneath the interface that impedes water penetration into the bulk.

**Figure 5:**
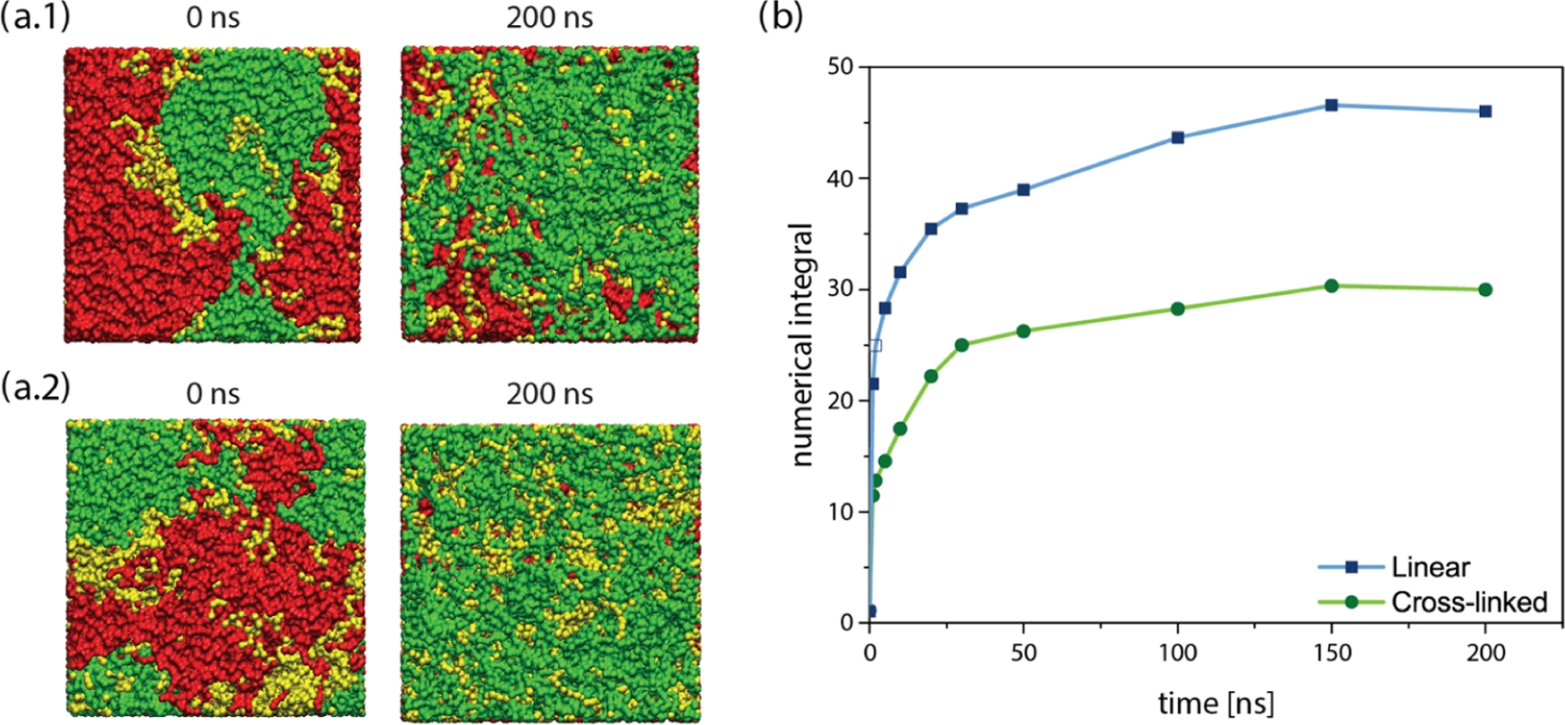
Top view of cross-linked (a.1) and linear (a.2) boxes before and after exposure to water. Numerical integral of PEG segments at the water interface (b).

A key difference in the interfacial morphology of cross-linked and linear polymers with water is the depth of penetration of water into the polymer. We quantified this depth by applying the sigmoid function (, *a* and *b* are constants) to the density profiles and calculated the thickness of the polymer–water interface layer, see the SI (Figure S9). Accordingly, the interface layer has 2.6 nm thickness for cross-linked topology and 4.8 nm thickness for linear topology, as shown in Figure 4, and this difference can be ascribed to the migration of more PEG chains in the case of linear topology due to the higher degree of flexibility of PEG segments as compared to the cross-linked polymer.

Figure 5 shows the top view of the simulation boxes at time 0 and 200 ns. We removed the water layer for better visualization of the (dry/wet) surface. As shown, most of the surface area is covered by a molecular layer of PEG, for both topologies (which is in line with similar wet film roughness, measured by AFM). However, the thicker PEG-rich interfacial layer is expected to better screen the underneath hydrophobic bulk (PC-rich region), resulting in an overall more hydrophilic polymer. We also quantified the time-resolved migration of PEG toward the interface by calculating the numerical integral of the PEG density at the interface over the duration of the simulations; see Figure 5b. Again, the considerably higher degree of PEG migration toward the interface for the linear polymer is clear, which could explain the significantly smaller WCA associated with this polymer topology. Also, the plateau region in the final 50 ns of the graphs indicates that the (200 ns) simulation time is sufficient to capture the steady state of the polymer–water interface rearrangements and there is no evident change in the surface as simulation time elapses.

According to MD simulations, PEG migrates toward the surface when polymers are exposed to water, causing higher surface hydrophilicity. However, the difference in the degree of hydrophilicity is due to the amount of PEG that migrated to the interface and the resulting thickness of the interface layer. It should be noted that the amount of water that penetrates into the interface layer has a significant impact on lowering the water contact angle. Thus, since PEG segments can move more freely in the case of a linear sample, a thicker interface layer is formed, allowing water to reach deeper layers into the polymer. The number of water molecules that penetrated the interface was 367100 for linear and 145400 for cross-linked samples. As a result, water comes in contact with a thicker layer of PEG soaked in water, which leads to the formation of a superhydrophilic surface as observed by WCA results.

## Conclusions

The effect of polymer topology on the amphiphilic polymer–water interface has been explored through a dual experimental/simulation approach on the molecular scale. Radically different wetting behaviors for (i) linear multiblock (WCA ≈ 0**°**) and (ii) cross-linked 3D network (WCA ≈ 50**°**) polyurethane coatings containing (hydrophobic) polycarbonate and/or (hydrophilic) PEG, with identical dry surface chemistry, was observed. This emphasizes the significant effect of polymer topology on wetting mechanisms. Real-time changes at the polymer–water interface were examined by AFM measurements and MD simulations, and it was discovered that PEG chains in both topologies migrate toward the surface and eventually cover a major part of the interface. Earlier WCA measurements of a PEG monolayer, i.e., WCA ≈ 30 **°**, indicate that PEG migration cannot fully explain superhydrophilic behavior, i.e., WCA ≈ 0**°**. The quantification of PEG migration to the polymer–water interface showed that the presence of more PEG causes the formation of a thicker interface layer in the case of linear polymer (4.8 nm) than the cross-linked network (2.6 nm). The increased thickness of the PEG-rich layer allows water to penetrate into the interface region and form (a thicker) highly hydrated layer, effectively shielding the underneath hydrophobic polycarbonate layers, and resulting in WCA ≈ 0°.

Previous studies have carefully established correlations between the morphological changes of soft diblock (amphiphilic) copolymers at the interface with water and polymer wetting process through theoretical^32,52^ and experimental^33,53^ approaches. In this work, we focused on the effect of topological constraints in the polymer structure on the interfacial morphological changes and the resulting wetting behavior of these coatings. Our findings demonstrate that the polymer architecture could significantly affect the polymer interfacial properties in submerged applications, which can be crucial for a variety of applications, e.g., antifouling, low-friction, and biomedical materials. In this work, we studied two extreme topological cases, i.e., linear (very flexible) and cross-linked (very constrained) structures. Nevertheless, as a future vision, employing (hyper)branched and dendritic architectures for amphiphilic multiblock polymers could provide a greater variety of polymer topologies to control the wetting properties.

## Supporting Information Available

Characterization of the synthesized polymers; coarse-grained parameters; simulation validations; characterization of the model polymers; contact angle images; XPS survey scans; DSC thermograms; AFM images of dry and wet linear polymer films; numerical density profiles of urethane groups; and sigmoid function fittings (PDF)
