# Supplementary material for: Molecular Insight into the Effect of Polymer Topology on Wettability of Block Copolymers: The Case of Amphiphilic Polyurethanes
Source: Langmuir. 2023 Dec 15;40(1):62–71. doi: 10.1021/acs.langmuir.3c01646 (PMC10786039; doi:10.1021/acs.langmuir.3c01646)
Supplement: Supplementary file 1 — la3c01646_si_001.pdf [file la3c01646_si_001.pdf]

Supporting Information for:

A molecular insight into the effect of polymer topology on wettability of block copolymers: case of amphiphilic polyurethanes

Alireza Mirzaalipour[1], Elnaz Aghamohammadi[1], Helma Vakili[2], Mohammadreza Khodabakhsh[3], Ugur Unal[3,4], Hesam Makki*[5]

[1]Department of Polymer and Color Engineering, Amirkabir University of Technology, 424 Hafez Ave., 159163-4311, Tehran, Iran.

[2]Polymer Engineering group, School of Chemical Engineering, College of Engineering, University of Tehran, 1417935840, Tehran, Iran.

[3]Chemistry Department, Koc University, Rumelifeneri yolu, Sariyer 34450 Istanbul, Turkey.

[4]Koc University Surface Science and Technology Center (KUYTAM), Koc University, Rumelifeneri yolu, Sariyer 34450 Istanbul, Turkey.

[5]Department of Chemistry and Materials Innovation Factory, University of Liverpool, Liverpool L69 7ZD, U.K.

Corresponding Author:

Hesam Makki

Email: hmakki@liverpool.ac.uk

1. Synthesized Polymers Characterization

Table S1. GPC molecular weights of the synthesized PUs.

Sample	M_n (g/mol)	M_w (g/mol)	*DI* (Mw/Mn)
**PEG 0%**	54883	74801	1.36
**PEG 10%**	56838	79166	1.39
**PEG 20%**	53765	73532	1.37
**PEG 30%**	51240	73056	1.43

2. Coarse-Grained (CG) Parameters

OPLS-AA (Optimized Potentials for Liquid Simulations) force field[1] was used to adjust atomistic MD simulations. The first step of MD simulations is energy minimization. 100 molecules of each material were minimized individually until the change in energy is less than 100 kJ/(mol)(nm). A V-rescale[2] thermostat was then used to run the simulation at a constant temperature (500 K) and constant volume (*NVT* step) for 500 ps with a 2-fs time step. The simulations were then performed with a 0.5 fs time step and 5 ns duration at *NPT* conditions (P = 1 bar, T = 500 K). A V-rescale thermostat and Parrinello-Rahman barostat[3] were utilized for this step. With a rate of 0.04 K/ps, cooling from 500 K to 300 K was finally completed. After cooling, the equilibrium was obtained under *NPT* (P = 1 bar, T = 300 K), similar to the initial *NPT* stage.

Equations S1 (bond) and S2 (angle) were used to describe the bonded interactions based on the Martini3 [4] method. The below described approach was used to determine equilibrium values and force constants for the bond and angle potentials. The distributions of the bonds and angles connecting the centers of volume of the atoms in a bead were first averaged over 5 ns trajectory data collected from the atomistic simulation. Equation S3 was used to calculate the potential values by converting the distribution of bonds and angles into probability. Equations S1 and S2 were fitted to potential-distance and potential-angle distributions, respectively, to derive the force constants for bonds and angles. The equilibrium values and force constants for all bonded interactions are provided in Tables S2 and S3. We ignored torsional potentials at the CG level.

$$U_{bond} = \frac{1}{2} K_{bond} (l - l_0)^2 \quad \text{(Eq-S1)}$$

$$U_{angle} = \frac{1}{2} K_{angle} [\cos(\theta) - \cos(\theta_0)]^2 \qquad \text{(Eq-S2)}$$

$$U = -\ln(p)\, kT \qquad \text{(Eq-S3)}$$

Table S2. Bond type parameters at CG level.

Bond	l_0 [nm]	K_{bond} [kJ/(mol)nm^2]
	PEG	
SN3r-SN3r	0.3119	7800
TP2a-SN3r	0.2692	11000
	PC	
TP2a-SC2	0.3051	7200
SC2-TC2	0.2830	6000
TC2-N2a	0.3086	14000
	HDI	
SP3a-TC2	0.2945	14000
TC2-TC2	0.2475	29500
	Cross-linker (HDI trimer)	
SP3a-TC2	0.2945	15500
TC2-TC2	0.2475	32000
TC2-SP3	0.2779	16400
SP3-SN2a	0.3180	13600
SN2a-TC2	0.2817	12500
	BD	
TP2a-TC2	0.2440	45500
	DMF	
TN2a-SN1a	0.2501	50000
	Urethane Linkage	
SP4-TN3	0.2900	30000

Table S3. Angle type parameterss at CG level.

Angle	Θ [degree]	K_{angle} [kJ/(mol)(rad^2)]
	PEG	
SN3r-SN3r-SN3r	121.43	35.5
TP2a-SN3r-SN3r	114.24	38.1
	PC	
TP2a-SC2-TC2	134.39	22.9
SC2-TC2-N2a	134.41	12.5
TC2-N2a-TC2	157.90	105.0
N2a-TC2-TC2	132.89	25.3
	HDI	
SP3a-TC2-TC2	120.15	240.0
TC2-TC2-TC2	148.75	470.5
	Cross-linker (HDI trimer)	

SP3a-TC2-TC2	119.73	241.6
TC2-TC2-TC2	147.50	474.8
TC2-SP3-SN2a	120.73	550.6
SP3-SN2a-SP3	74.00	122.0
SP3-SN2a-TC2	136.12	197.8
TC2-TC2-SN2a	163.94	152.5

3. Simulation Validations

By comparing the CG level densities with experimental and atomistic level data, the validity of the bonded and non-bonded interactions was evaluated. The end-to-end distance and radius of gyration for polymer chains were also compared at the atomistic and CG levels, see Table S4.

Table S4. A comprehensive comparison betweeen exprimental, atomistic and CG level simulations properties.

**Materials**	**Density [g/cm$^{3]}$**			**radius of gyration [nm]**		**end-to-end distance [nm]**	
	Experimental	Atomistic	CG Martini	Atomistic	CG Martini	Atomistic	CG Martini
**Cross-linker**	1.14	1.06	1.07	-	-	-	-
**DMF**	0.95	0.92	1.01	-	-	-	-
**BD**	1.02	1.02	0.88	-	-	-	-
**HDI**	1.05	1.00	0.94	-	-	-	-
**PC**	1.09	0.92	1.11	1.73	1.79	4.13	4.43
**PEG**	1.21	1.13	1.25	1.06	1.38	2.31	3.03

4. Model Polymers Characterization

Table S5. Linear samples molecular weights estimated by molecular dynamics simulation.

Sample	M_n (g/mol)	M_w (g/mol)	*DI* (Mw/Mn)
**PEG 0%**	29400	42900	1.5
**PEG 30%**	40800	92300	2.3

Table S6. Molecular weight between crosslinks (Mc) cross-link density based on molecular dynamics simulation.

Sample	M_c (g/mol)	Cross-link density (mol/m^3)
**PEG 0%**	3464	292
**PEG 30%**	3349	308

5. ATR-IR

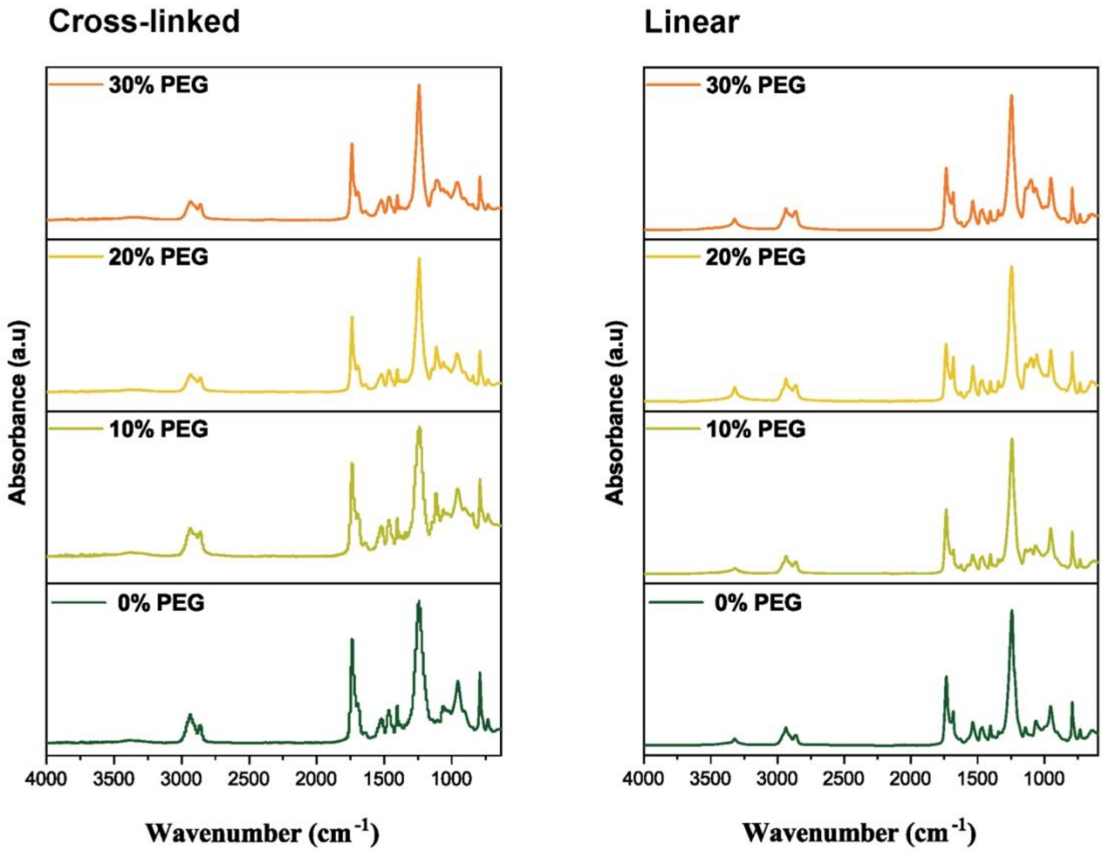

Figure S1. ATR-FTIR spectra of cross-linked and linear samples containing different amount of PEG. Note that PEG percentage shows the amount of PC replaced by PEG in the formulation, see Tables 1 and 2.

6. Contact Angle

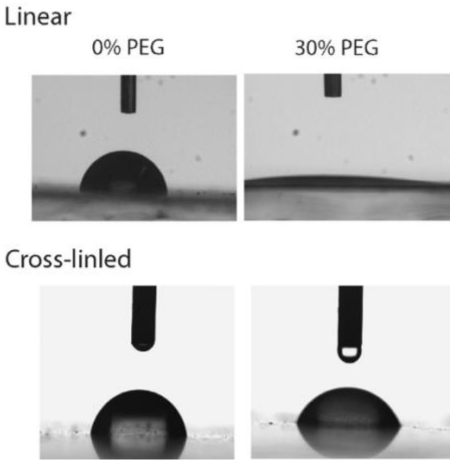

Figure S2. Contact angle images of linear and cros-lined samples.

6. XPS Survey Scan

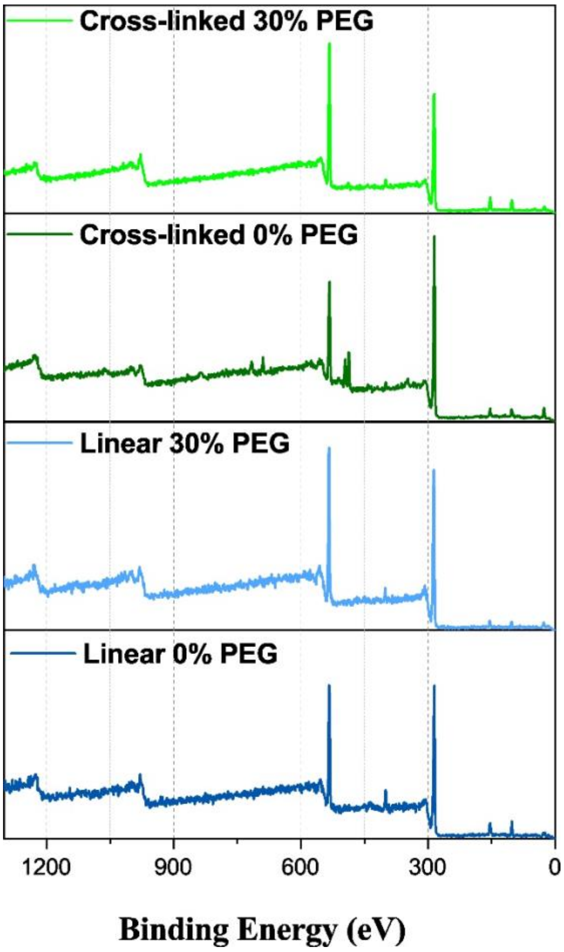

Figure S3. XPS survey scans of linear and cross-linked PEG 0% and PEG 30% samples.

A peak around 490 eV is discernible for cross-linked 0% sample. This peak can be detected in all samples and attributed to dibutyltin dilaurate ((CH3(CH2)10CO2)2Sn((CH2)3CH3)2), the Sn band, which we used as the catalyst. However, the stronger peak for the cross-linked 0% PEG sample, might be caused by the excessive amount of catalyst utilized in its synthesis.

7. DSC Measurement

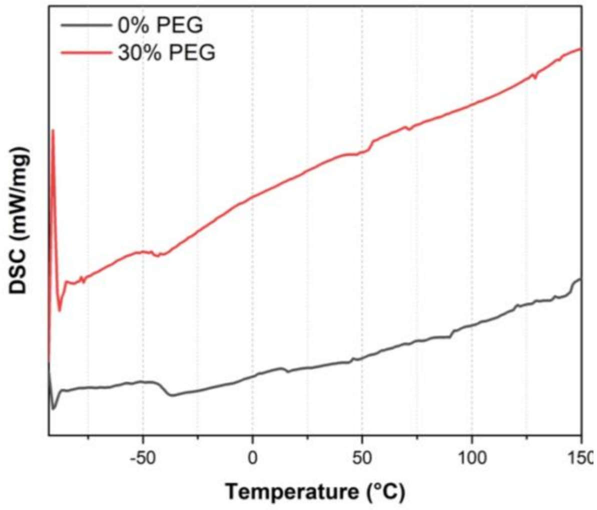

Figure S4. DSC thermograms of the cross-linked PEG 0% and PEG 30% samples.

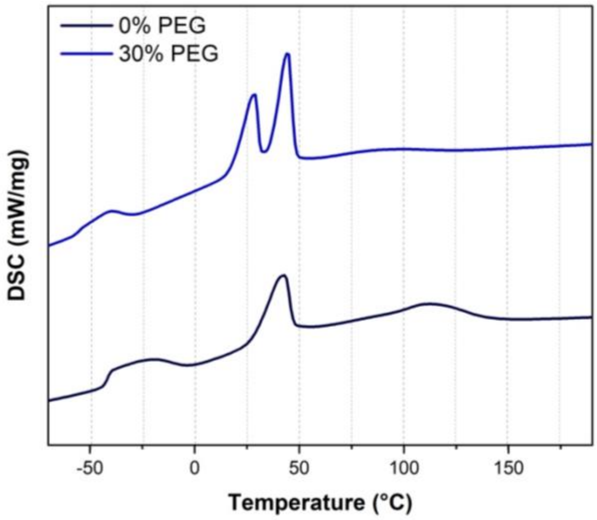

Figure S5. DSC thermograms of the linear PEG 0% and PEG 30% samples.

Table S7. Meltig point and T_g values for different topologies of PEG 30% samples.

Sample	$T_{m,PC}$	$T_{m,PEG}$	T_g
Cross-linked	-	-	50.6, -47
Linear	44	27	102.2, -31.1

8. AFM measurements

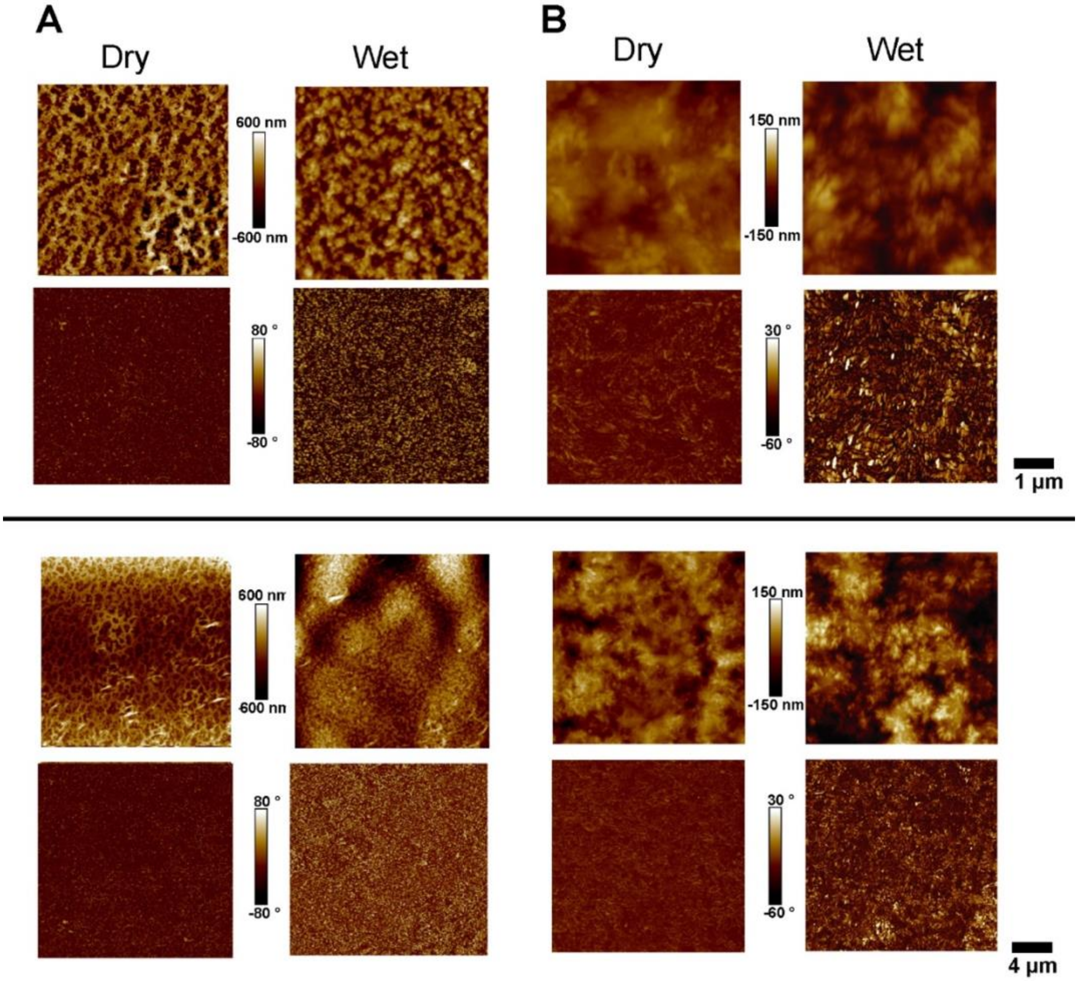

Figure S6. AFM topographical and phase images of 0% PEG (a) and 30% PEG (b) cross-linked PUs the images for two measurement scan sizes (5x5 and 20x20 µm) are shown.

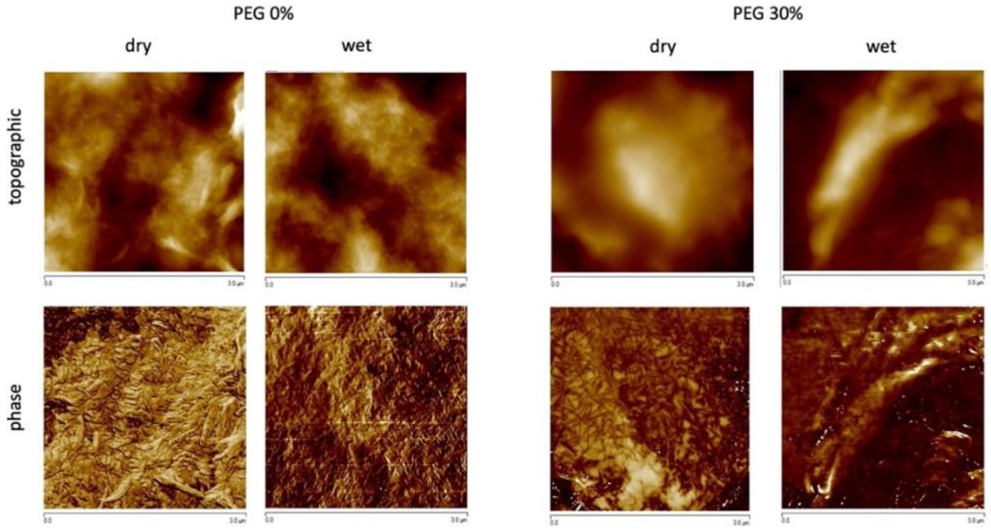

Figure S7. AFM topography and phase images of dry and wet linear PEG 0% and 30% samples.

9. Numerical density profiles of urethane groups

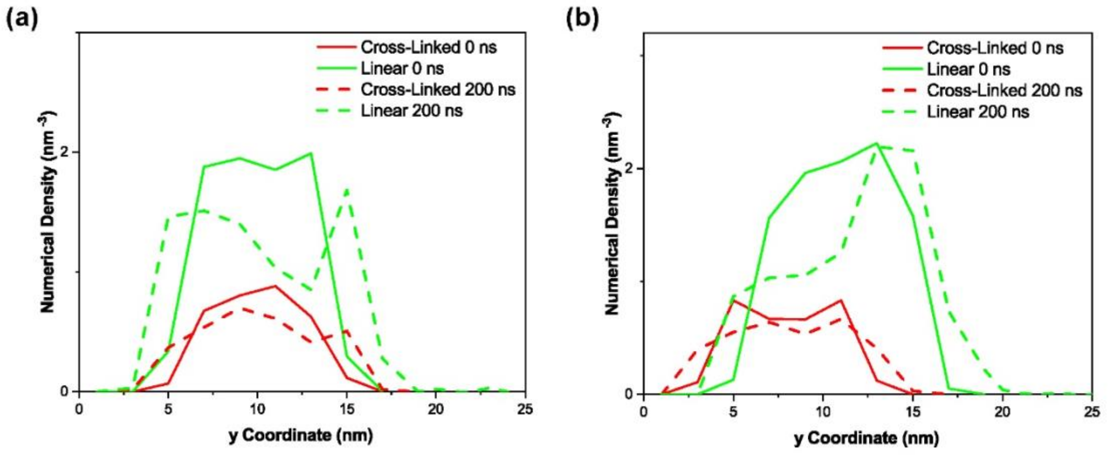

Figure S8. Numerical density profiles of urethane groups in PEG 0% (a) and PEG 30% (b) samples.

10. Sigmoid function fitting

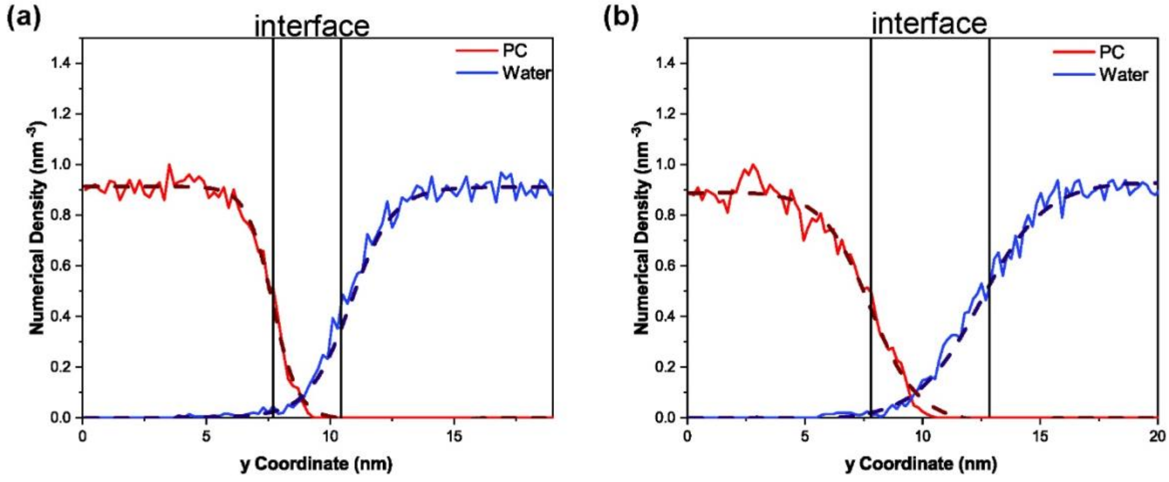

Figure S9. Numerical densities of PC and water at interface and Sigmoid function fitting (dashed lines) for cross-linked (a) and linear (b) sample .

References

[1] W. L. Jorgensen, D. S. Maxwell, and J. Tirado-Rives, ‘Development and Testing of the OPLS All-Atom Force Field on Conformational Energetics and Properties of Organic Liquids’, *J Am Chem Soc*, vol. 118, no. 45, pp. 11225–11236, Nov. 1996, doi: 10.1021/ja9621760.

[2] G. Bussi, D. Donadio, and M. Parrinello, ‘Canonical sampling through velocity rescaling’, *J Chem Phys*, vol. 126, no. 1, p. 014101, Jan. 2007, doi: 10.1063/1.2408420.

[3] M. Parrinello and A. Rahman, ‘Polymorphic transitions in single crystals: A new molecular dynamics method’, *J Appl Phys*, vol. 52, no. 12, pp. 7182–7190, Dec. 1981, doi: 10.1063/1.328693.

[4] P. C. T. Souza, R. Alessandri, J. Barnoud, S. Thallmair, I. Faustino, F. Grünewald, I. Patmanidis, H. Abdizadeh, B. M. H. Bruininks, T. A. Wassenaar, P. C. Kroon, J. Melcr, V. Nieto, V. Corradi, H. M. Khan, J. Domański, M. Javanainen, H. Martinez-Seara, N. Reuter, R. B. Best, I. Vattulainen, L. Monticelli, X. Periole, D. P. Tieleman, A. H. de Vries, and S. J. Marrink, ‘Martini 3: a general purpose force field for coarse-grained molecular dynamics’, *Nat Methods*, vol. 18, no. 4, pp. 382–388, 2021, doi: 10.1038/s41592-021-01098-3.
